# Uterine Tissue Metabonomics Combined with 16S rRNA Gene Sequencing To Analyze the Changes of Gut Microbiota in Mice with Endometritis and the Intervention Effect of Tau Interferon

**DOI:** 10.1128/spectrum.00409-23

**Published:** 2023-04-17

**Authors:** Guanhong Xue, Zhijie Zheng, Xiaoben Liang, Yonghui Zheng, Haichong Wu

**Affiliations:** a Department of Veterinary Medicine, College of Animal Sciences, Zhejiang University, Hangzhou, Zhejiang, People’s Republic of China; Jilin University

**Keywords:** IFN-τ, microbiota, metabolomics, gut microbiota, endometritis

## Abstract

Endometritis is a common cow disease characterized by inflammation of endometrium, which leads to infertility or low fertility of cows and brings huge economic losses to the dairy industry. Tau interferon (IFN-τ) has many important biological functions, including an anti-inflammatory effect. The present study aimed to survey the effects of IFN-τ administration on gut microflora and body metabolism in mice with endometritis and to explore the potential relationship. The results indicated that IFN-τ obviously alleviated the damage and ultrastructural changes of mouse endometrium induced by Escherichia coli and enhanced tight junction protein’s expression level. Through analysis by 16S rRNA gene sequencing, we found that IFN-τ, especially at 12 h, could regulate the composition of gut microbiota associated with *Pediococcus*, Staphylococcus, and *Enterorhabdus* in E. coli-induced mouse endometritis. Through histometabonomics, it was found that endometritis was related to 11 different metabolites and 4 potential metabolic pathways. These metabolites and metabolic pathways were major participants in metabolic pathways, cysteine and methionine metabolism, arachidonic acid metabolism, and pyrimidine metabolism. Correlation analysis of gut microbiota with uterine tissue metabolomics showed that changes in metabolic pathways might be affected by gut microbiota, such as *Enterorhabdus* in mouse endometritis. The above results indicated that the anti-inflammatory mechanism of IFN-τ might be reduction of the abundance of *Enterorhabdus* in the gut microbiota, affecting the expression level of important metabolites in uterine tissue and thus playing an anti-inflammatory role.

**IMPORTANCE** The change in intestinal flora has been the focus of many disease studies in recent years, but the pathogenetic effect of interferon on endometritis is still unclear. The results of this study showed that IFN-τ alleviated the damage in mouse endometritis induced by *E. coli* and improved the endometrial tissue barrier. Its functional mechanism may be reduction of the abundance of *Enterorhabdus* in the intestinal microbiota, affecting the expression level of important metabolites in uterine tissue and thus playing an anti-inflammatory role.

## INTRODUCTION

Endometritis is an inflammation of the endometrium, which leads to infertility or low fertility in dairy cows by affecting the function of the uterus and ovary (1, 2). At present, a serious worldwide economic problem facing the cattle industry is bovine endometritis, because it is closely related to the reduction of reproductive activities, resulting in the increase of farm costs (3). Studies have shown that 80% to 90% of cow endometritis is caused by pathogen infection, which is characterized by decay and moisture and reddish brown uterine secretion, accompanied by fever, dehydration, and depression (4, 5). Escherichia coli, a common Gram-positive bacterium, is considered the main microorganism associated with bovine endometritis (6). It stimulates the immune defense system of the uterus, leading to inflammation and endometrial tissue damage (7). Due to the infectivity, impact on animal health, and potential threat to life, antibiotics are considered beneficial for the treatment of endometritis. However, each use of antibiotics is related to the selective pressure toward drug-resistant bacteria (8, 9). Obviously, the increase of antibiotic resistance is one of the reasons for the decrease of clinical efficacy and may harm animal welfare and aggravate economic losses (10). Therefore, new treatments need to be developed to treat bovine endometritis.

Currently, the gut microbiota is a research hot spot in many scientific fields, being found to act as a key character in the development of infectious diseases (11). The imbalance of gut microbiota could lead to disorders of the intestinal barrier and bacterial translocation, triggering a persistent state of systemic inflammation and resulting in the onset and progression of the disease (12). Besides, gut microbiota serves as a bioreactor for metabolic and immunological functions which can regulate the response to external stimuli in the host environment (13). The effect of gut microbiota on host metabolism has been reported in many diseases with intestinal dysbacteriosis (13, 14). However, there are few reports on the association between gut microflora and host metabolism in endometritis.

Tau interferon (IFN-τ) is a member of the type I interferon family and shows multiple immunomodulatory properties (15). Previous researches have indicated that IFN-τ has a strong anti-inflammatory effect and a broad cross-species reactivity in humans and other animals (16–18). In addition, the pleiotropic effects of IFN-τ, including stimulating amino acid transport and metabolism, contribute to animal survival, growth, and development (19). Nevertheless, there are very few reports on whether the variety in tissue metabolism and microbiota diversity are the cause or consequence of endometritis and the intervention effect of IFN-τ. From the perspective of microbiomics and metabolomics, it is very important to clarify the characteristics of endometritis for understanding its pathogenesis and potential threat.

This study was intended to investigate the influence of IFN-τ administration on gut microflora and body metabolism in mice with endometritis and to explore the potential relationship. 16S rRNA sequencing and liquid chromatography-mass spectrometry (LC-MS) were performed to analyze the differences of intestinal microbial community structure and tissue metabonomics. The present study supplied a more comprehensive and detailed understanding of the mechanism by which IFN-τ mediates a therapeutic effect in E. coli-induced endometritis through regulating the host gut microbiota.

## RESULTS

### Effects of IFN-τ on E. coli-induced pathological changes.

**FIG 1:**
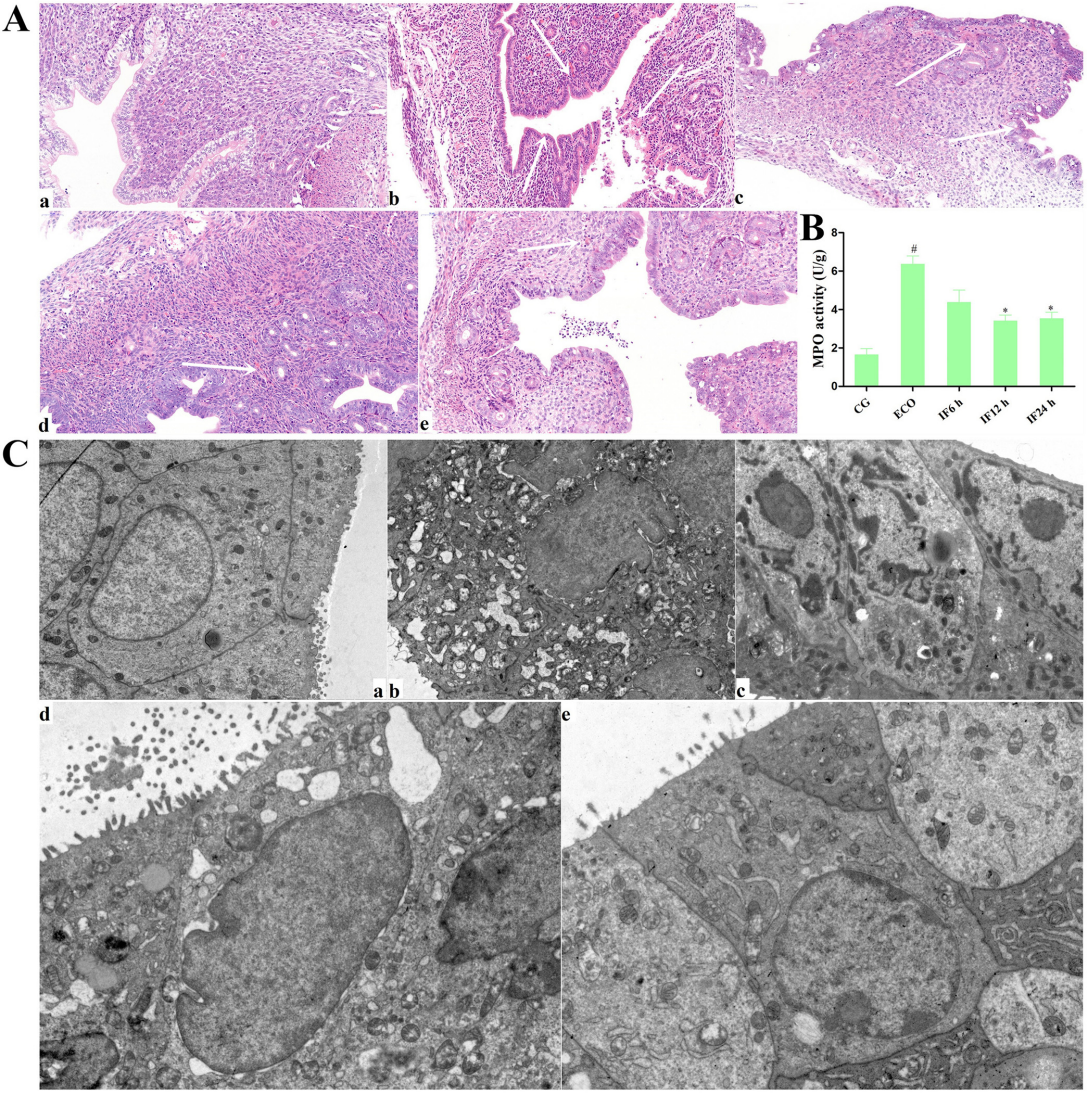
Effects of IFN-τ on E. coli-induced pathological changes. (A) (a) Control group; (b) ECO group; (c to e) IFN-τ 6-, 12-, and 24-h treatment groups, respectively (bar, 50 μm). The white arrow represents the location of tissue damage. (B) MPO activity. (C) The ultrastructure of mouse endometrium was observed by TEM (the lowercase letters a to e represent the same groups as in panel A). Data are presented as the mean ± SEM from three independent experiments. *, significant difference at *P* < 0.05; #, *P* < 0.05 versus Control group.

The morphology of the uterus in the control group was intact without pathological changes according to histological analysis (Fig. 1A, a). However, the uterus in the E. coli group was severely damaged with extensive inflammatory cell infiltration (Fig. 1A, b). In contrast, the infiltration of inflammatory cells in the IFN-τ group was reduced, and the endometrial structure was relatively intact (Fig. 1A, c to e). As shown in Fig. 1B, IFN-τ treatment significantly reduced *E. coli*-induced myeloperoxidase (MPO) activity, especially in the 12-h group.

Ultrastructural images of mouse endometrium using transmission electron microscopy (TEM) showed nuclear chromatin marginalization, agglomeration, aggregation, mitochondrial vacuoles, and endoplasmic reticulum swelling in the E. coli group. However, treatment with IFN-τ dramatically alleviated morphological lesions. The integrity of mouse endometrium in the control group was maintained (Fig. 1C).

### IFN-τ enhanced the barrier of mouse endometrium induced by E. coli.

**FIG 2:**
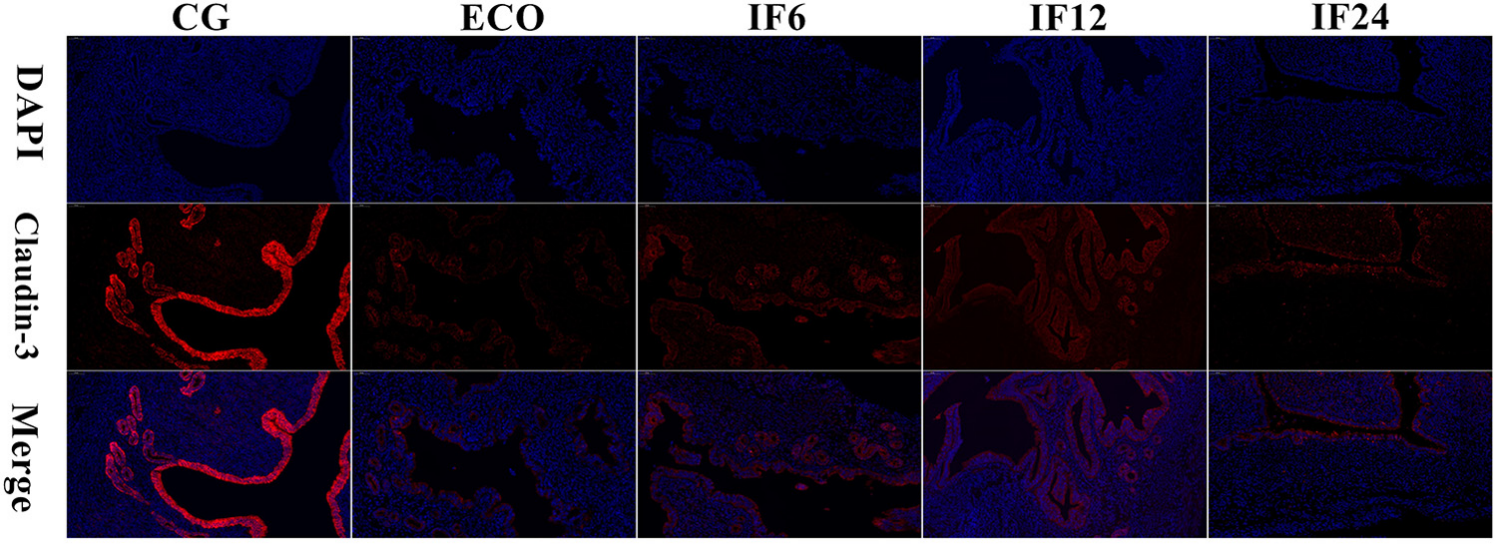
IFN-τ enhanced the barrier of mouse endometrium induced by E. coli. The expression of tight junctional protein claudin-3 was determined by immunofluorescence.

The tight junction proteins are located on the top lateral membrane and are necessary to maintain the mucosal barrier. An immunofluorescence assay was used to evaluate the integrity of mouse endometrium barrier (Fig. 2). The result showed that IFN-τ significantly increased the expression of tight junction protein claudin-3 inhibited by *E. coli*.

### Reduced bacterial diversity in fecal microbiome associated with endometritis.

In the current microbiome survey, we obtained 2,742,316 reads, of which 82.59% passed the quality check. Of the cleaned reads, 65.82% (1,805,032) were joined. Effective tags were divided into operational taxonomic units (OTUs) according to 97% similarity. The species accumulation curve (see Fig. S1 in the supplemental material) and rarefaction curve (Fig. S2) of all samples demonstrate the sufficiency of sampling.

**FIG 3:**
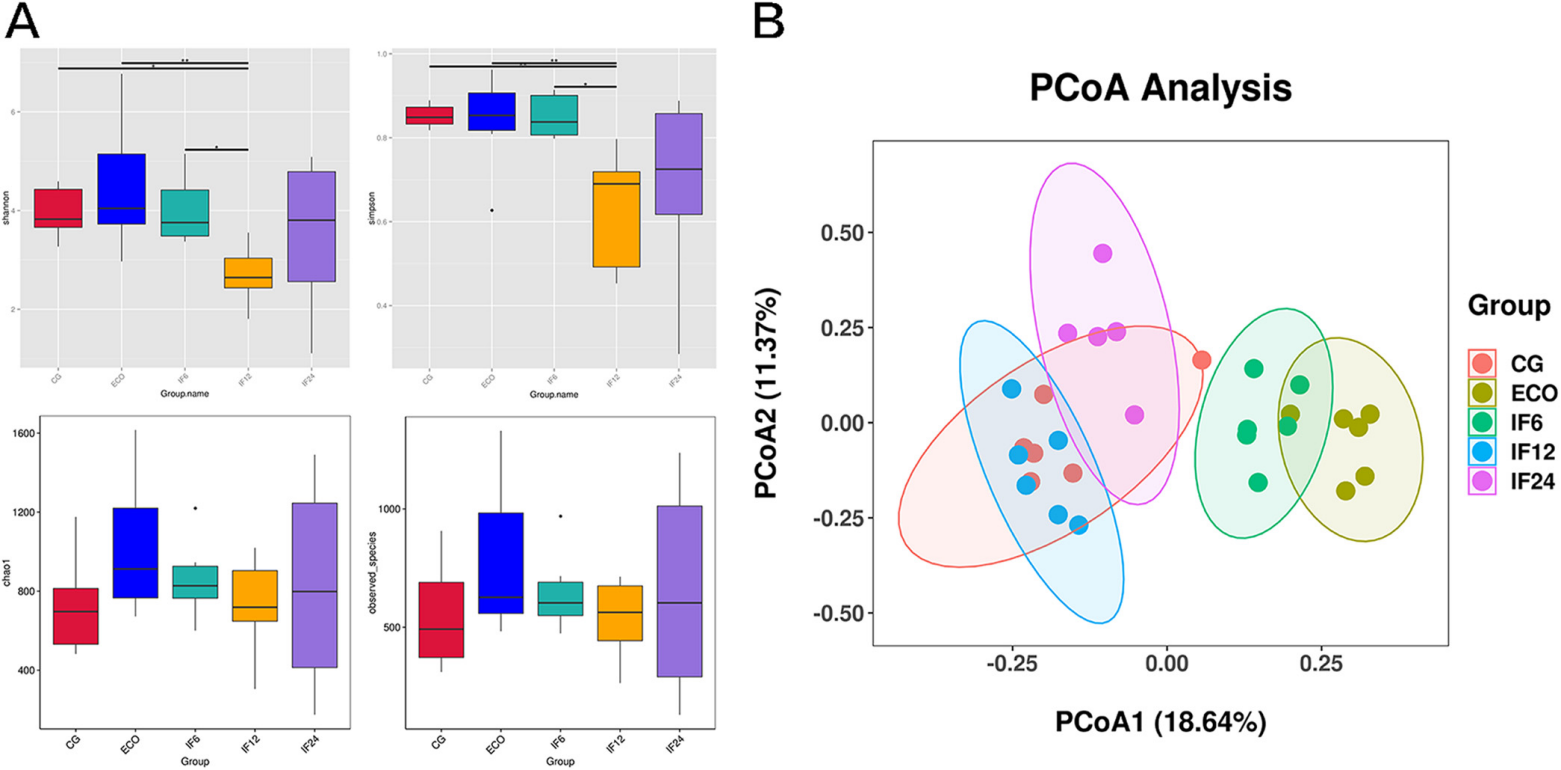
Gut microbiome diversity and structure analysis. (A) Species diversity differences among five groups were estimated by the observed species, Chao1, Shannon, and Simpson indices. (B) PCoA plots of bacterial β diversity based on Bray-Curtis metrics. * and **, significant differences at *P* < 0.05 and *P* < 0.01, respectively.

In order to estimate the differences in bacterial diversity among these groups, sequences were compared for evaluation of α diversity and β diversity. In α diversity analysis, Chao1 and observed species were used to evaluate the bacterial community abundance, Shannon and Simpson analyses were performed to calculate community diversity. Obviously, statistics have differences in Shannon (*P* = 0.01) and Simpson (*P *= 0.0071) indices between the ECO and IF12 groups, but there were no significant differences between Chao1 and the observed species between all groups (Fig. 3A). Through principal-coordinate analysis (PCoA), we can preliminarily find that although the bacterial community abundances of each group are similar, there are differences in community diversity, especially between the ECO group and IF12 group.

To extract the most important elements and structures from multidimensional data, PCoA was performed on five groups of fecal samples according to the Bray-Curtis difference. As shown in Fig. 3B, PCoA 1 and 2 accounted for 18.64% and 11.37% of the total change, respectively. PCoA 1 could observably separate microbial communities in fecal samples from CG, ECO, and IF12 groups. What stands out in the figure is that the difference between ECO and IF12 groups is significant. It is apparent from the above results that the diversity of gut microbiota can be significantly affected by IFN-τ, especially by treatment at 12 h.

### Alterations in the composition of fecal microflora associated with endometritis.

**FIG 4:**
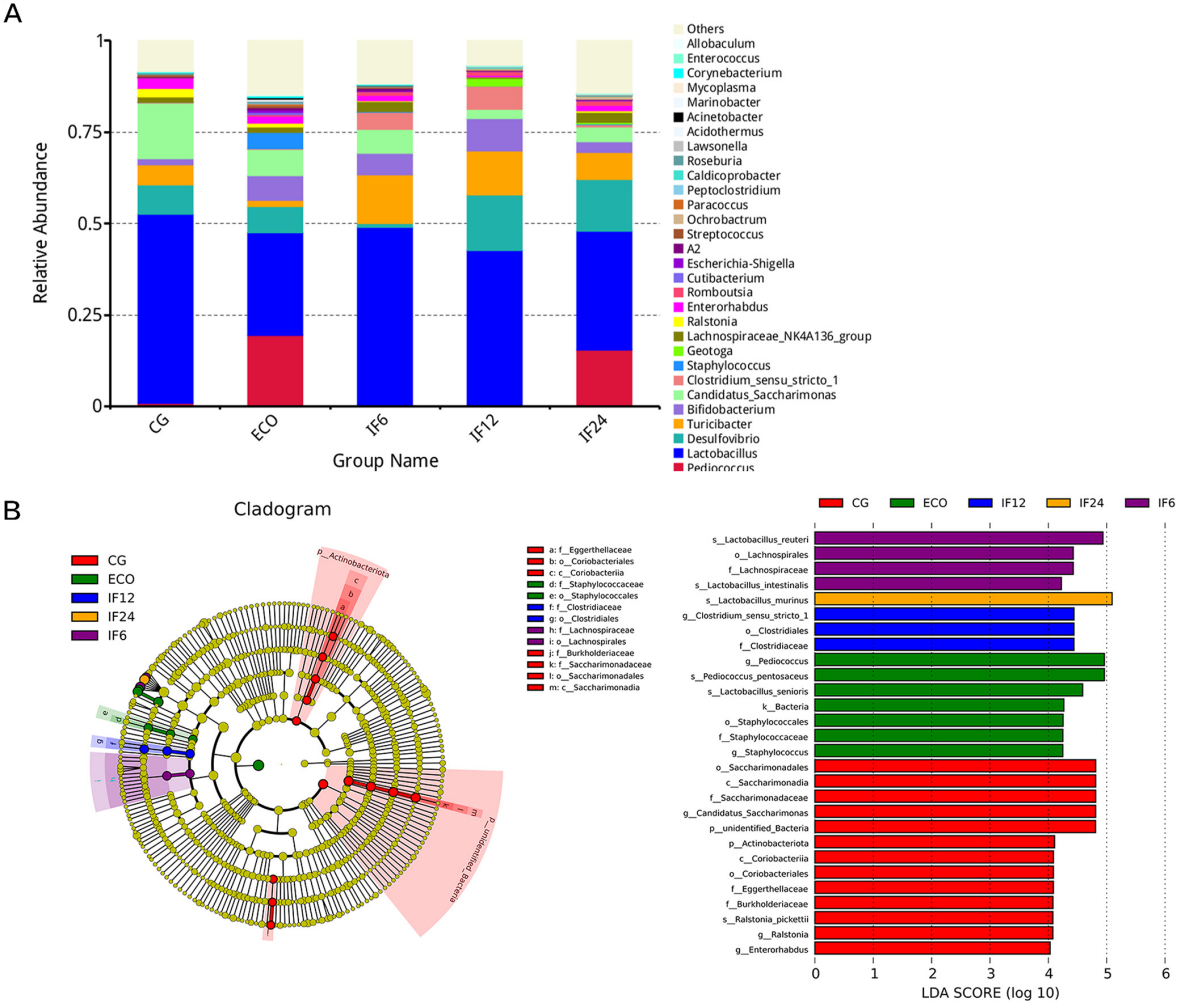
Alterations in the composition of fecal microflora associated with endometritis. (A) Component proportion of bacterial genus in each group. (B) Linear discriminant analysis (LDA) integrated with effect size (LEfSe).

The relative proportions of dominant taxa at the genus level were estimated by the distribution of microbial taxa in these groups. Significant differences exist in the gut microbiota across samples among groups (Fig. 4A). Thirty genera were identified for each group. *Lactobacillus* was the primary genus, accounting for 51.8% in the CG group, 28.1% in the ECO group, 48.7% in the IF6 group, 42.7% in the IF12 group and 32.7% in the IF24 group. Besides, *Pediococcus* was enriched not only in the ECO group compared to the CG group (19.7% versus 7%) but also in the ECO group compared to the IF12 group (19.7% versus 5%). It should also be worthy of attention that the percentage of Staphylococcus in the ECO group (4.5%) was higher than that in the CG (0.7%) and IF12 (0.3%) groups. Furthermore, the percentage of *Enterorhabdus* also had an obvious difference between ECO (1.9%) and IF12 (0.4%) groups.

Linear discriminant analysis (LDA) effect size (LEfSe) analysis based on rank test was carried out to generate a branching map to distinguish the specific bacteria related to endometritis. LEfSe analysis distinguished the intestinal microbial communities of each group by an LDA score of >4 (Fig. 4B). At the genus level, *Pediococcus* was obviously increased in the ECO group compared with the CG group, while *Pediococcus* was obviously decreased in the IF12 group compared with the ECO group. Compared with the CG group, Staphylococcus in the ECO group was significantly higher, while Staphylococcus in the IF12 group was obviously lower than that in the ECO group.

Collectively, these facts and data may be a convincing proof that the occurrence of endometritis is associated with gut microbiota. In other words, IFN-τ regulated the gut microbiota composition in mouse endometritis to intervene in the inflammatory response caused by E. coli, especially at 12 h.

### Metabolite changes associated with endometritis.

Subsequently, the metabolite analysis of uterine samples was performed by a liquid chromatography-electrospray ionization-tandem mass spectrometry (LC-ESI-MS/MS) system. The total ion chromatogram (TIC) of the uterine tissue is shown in Fig. S3. There were 598 metabolites identified in the uterine tissue including 123 amino acid and metabolomics, 72 nucleotide and metabolomics, 82 organic acid and derivatives, 32 carboxylic acids and derivatives, 48 oxidized lipids, 87 fatty acids (FA), and 154 others (Table S1). OPLS-DA (orthogonal partial least-squares discriminant analysis) provides a multivariate statistical analysis method which has supervised pattern recognition and maximizes the differences between groups, enabling one to find different metabolites. OPLS-DA score plots of metabolites are shown in Fig. S4. The results displayed great difference between ECO and IF12 groups in metabolite composition.

### Differential metabolite screening and analysis.

**FIG 5:**
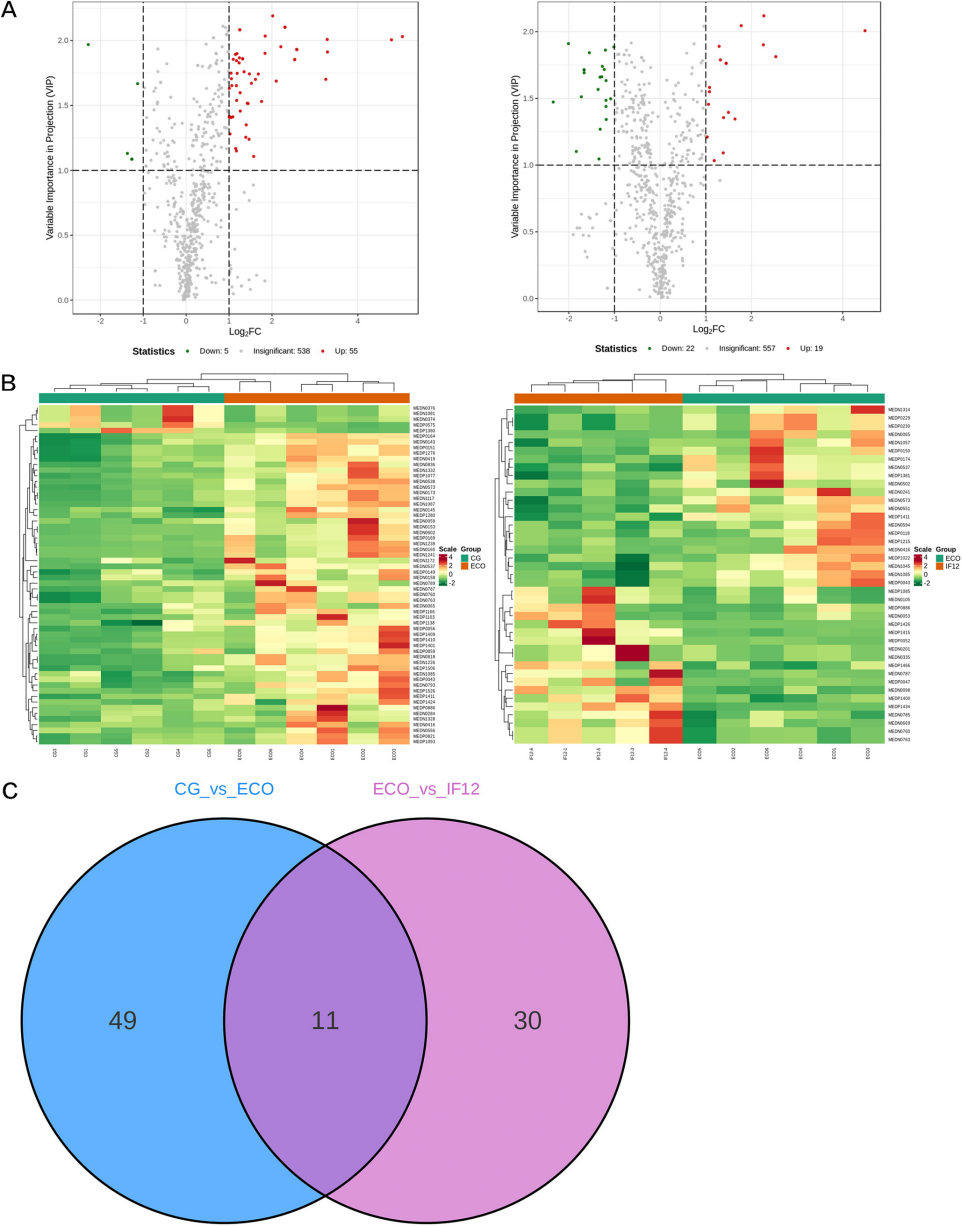
Differential metabolite screening and analysis in CG group versus ECO group (left) and in ECO group versus IF12 group (right). (A) Volcano plots show the expression levels of different differential metabolites in CG group versus ECO group and in ECO group versus IF12 group. The green dots represent downregulated differentially expressed metabolites, the red dots represent upregulated differentially expressed metabolites, and the black dots represent metabolites that are detected but not significantly different. (B) Heatmap visualization. The profiles of all types of differential metabolites in CG group versus ECO group and in ECO group versus IF12 group were normalized to complete the linkage hierarchical clustering. Each sample was represented by one column, and each metabolite is visualized in one row. (C) Venn diagram showing the overlapping and accession-specific differential metabolites.

We further screened differential metabolites among CG, ECO, and IF12 groups according to fold changes (FC of ≥2 or ≤0.5) and variables identified as important in the prediction (VIP of >1) scores. The filtered results are shown in Fig. 5; there were 60 differential metabolites (5 downregulated and 55 upregulated) in the CG group compared to the ECO group and 41 differential metabolites (22 downregulated and 19 upregulated) in the ECO group compared to the IF12 group. However, 11 differential metabolites were identified in both CG-versus-ECO group and ECO-versus-IF12 group comparisons. Compared to the CG group, differential metabolites such as *O*-phospho-l-serine, ureidoisobutyric acid, ADP-ribose, GDP-l-fucose, glutathione oxidized, and carnitine C_13:0_ were upregulated in the ECO group. Notably, compared with the ECO group, the above six differential metabolites showed a contrasting trend in the IF12 group (Table 1). It was obvious that these differential metabolites were regarded as important metabolites of IFN-τ intervention in mice with endometritis.

**TABLE 1:** Differential metabolites in CG group versus ECO group and in ECO group versus IF12 group

Index	Metabolite	VIP		Fold change		CG vs ECO	ECO vs IF12
		CG vs ECO	ECO vs IF12	CG vs ECO	ECO vs IF12		
MEDN0065	*O*-Phospho-l-serine	1.35	1.51	2.64	0.30	Up	Down
MEDN0416	Ureidoisobutyric acid	1.11	1.47	2.98	0.19	Up	Down
MEDN0537	ADP-ribose	2.19	1.86	4/05	0.44	Up	Down
MEDN0573	GDP-l-fucose	1.95	1.69	4.61	0.32	Up	Down
MEDP0043	Glutathione oxidized	1.65	1.49	2.25	0.44	Up	Down
MEDP1411	Carnitine C_13:0_	1.85	1.84	2.26	0.34	Up	Down

### Differential metabolic pathway enrichment analysis.

**FIG 6:**
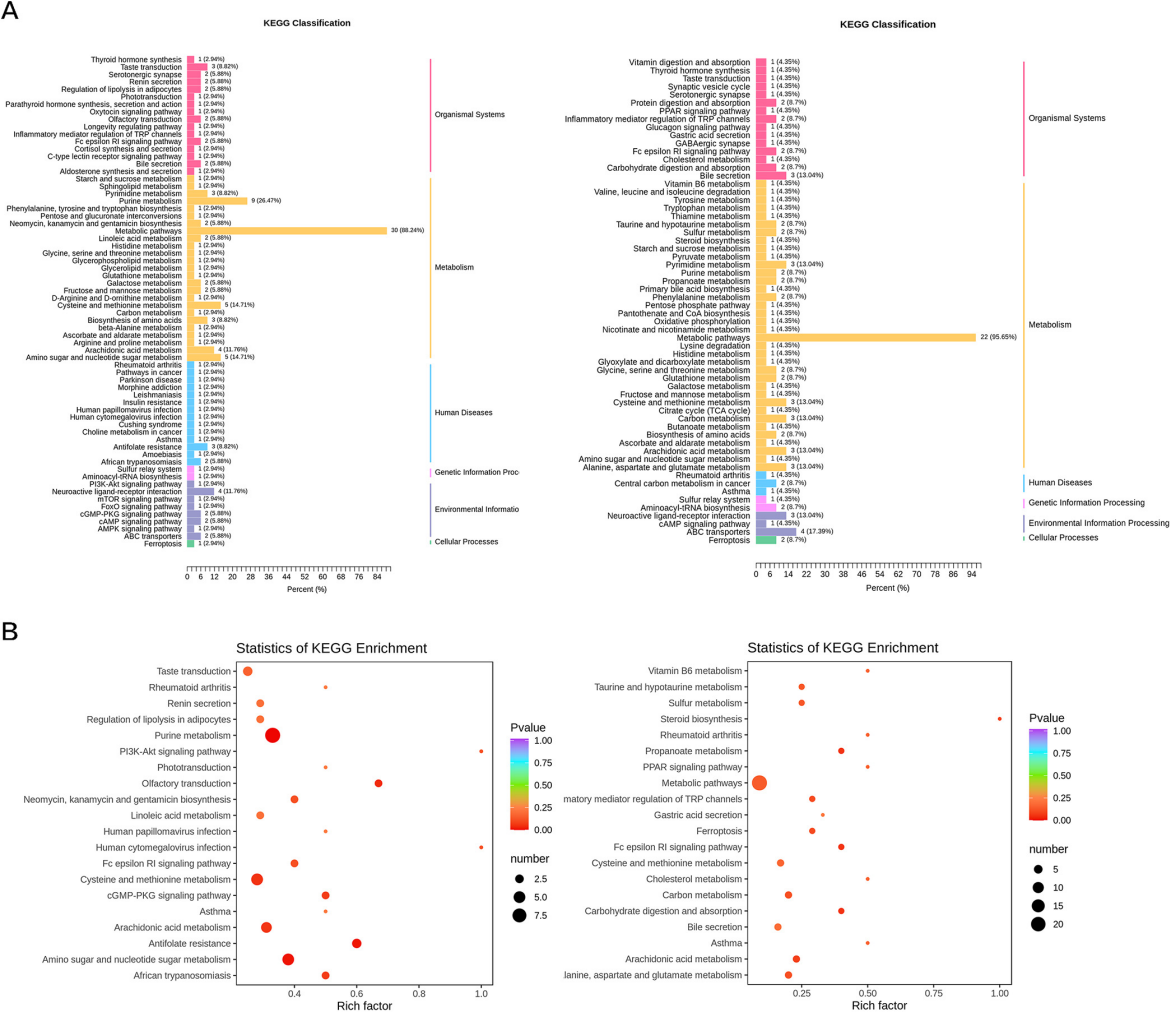
KEGG functional annotation and enrichment analysis of differential metabolites. (A) KEGG classification of differential metabolites in CG group versus ECO group and in ECO group (left) versus IF12 group (right). (B) KEGG enrichment of differential metabolites in CG group versus ECO group (left) and in ECO group versus IF12 group (right). Rich factor is the ratio of the number of differentially expressed metabolites in the corresponding pathway to the total number of metabolites detected by the pathway. The higher the value, the greater the enrichment degree. The closer the *P* value is to 0, the more significant the enrichment. The size of the dots represents the number of differentially significant metabolites enriched to the corresponding pathway. Abbreviations: PI3K, phosphatidylinositol 3-kinase; AMPK, AMP-activated protein kinase; PPAR, peroxisome proliferator-activated receptor; CoA, coenzyme A; TCA, tricarboxylic acid; PKG, protein kinase G; TRP, transient receptor potential.

We mapped the differential metabolites between the five groups to the KEGG for detailed pathway information (Fig. 6). There existed 66 differential metabolic pathways between the CG group and the ECO group and 60 metabolic pathways between the ECO group and the IF12 group. The mutual differential metabolic pathways between CG and ECO groups and between ECO and IF12 groups were mainly participants in the metabolic pathways of cysteine and methionine metabolism, pyrimidine metabolism, and arachidonic acid metabolism, which might be the essential metabolic pathways associated with IFN-τ intervention in mice with endometritis.

### Correlation analysis.

**FIG 7:**
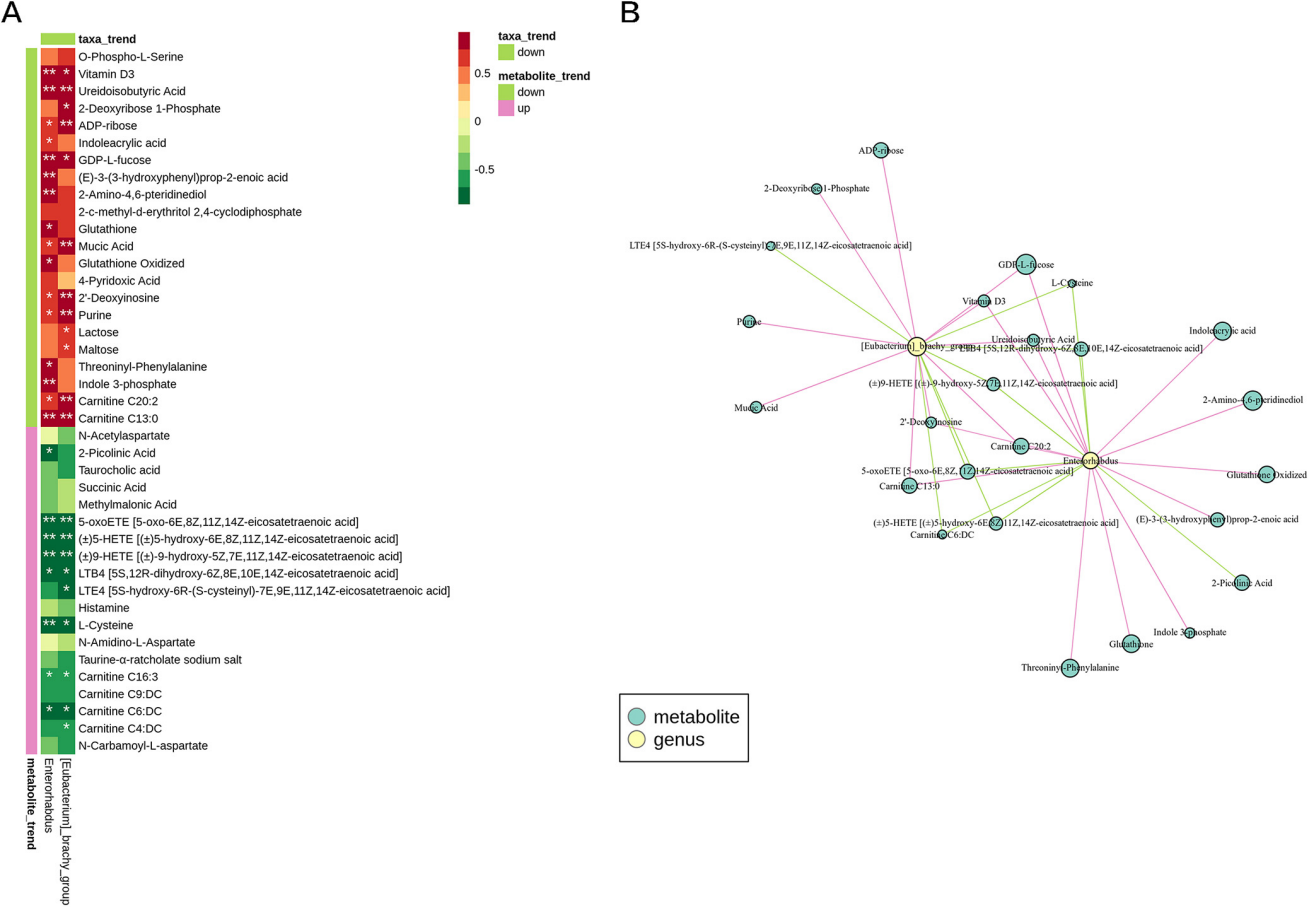
Correlation analysis between gut microbes and uterine metabolites in ECO and IF12 groups. (A) Correlation heatmap shows the degree of correlation between differential metabolites and differential gut microbes. (B) Network diagram shows the relationship between microbes and metabolites.

Based on the selection of microorganisms and metabolites with a significant correlation test *P* value of ≤0.05 and a correlation of >0.7, a correlation heatmap and a network diagram were used to show the covariates between gut microorganisms and uterine metabolites in ECO and IF12 groups. As shown in Fig. 7, *Enterorhabdus* was related to a number of metabolites, including ureidoisobutyric acid, ADP-ribose, GDP-l-fucose, glutathione oxidized, and carnitine C_13:0_. Besides, there is a positive correlation between *Enterorhabdus* and the above five metabolites. These results were mutually verified with the above gut microbe analysis results and were consistent with the existing results in uterine metabolite analysis. The results suggested that IFN-τ might reduce the above metabolisms by inhibiting the abundance of *Enterorhabdus* and finally achieving its anti-inflammatory effect.

## DISCUSSION

Endometritis is an inflammation of the endometrium after parturition, caused by an infection with invasive microorganisms (20). It not only influences the normal fertility of cows but also brings about great economic losses to the livestock industry (21). At present, antibiotics are the main treatment for most cow endometritis, but their disadvantages, such as the increase of bacterial resistance and drug residues, outweigh their advantages (22, 23). It has been previously shown that IFN-τ has an anti-inflammatory property (16, 17, 24). In the present study, IFN-τ significantly alleviated the damage and ultrastructural changes of mouse endometrium induced by E. coli and enhanced the expression level of tight junction protein. The tight junction proteins are located on the top lateral membrane and are necessary to maintain the mucosal barrier (25). However, few studies to date have examined how they interact with the microbiota diversity and tissue metabolism.

More and more evidence has shown that the diversity of gut microbiota can produce a wide range of small molecules (such as metabolites), which are related to many important pathways such as energy homeostasis, nutrient intake, and immune balance (26, 27). Although the latest research suggests that the imbalance of gut microbiota could lead to a continuous state of systemic inflammation, the possible relationship between gut microbiota and host metabolism in endometritis remains unclear. The 16S rRNA gene sequencing suggested that the richness of gut microbiota could be changed by E. coli. Compared to the ECO group, the CG and IF12 groups showed great differences in gut microbiota. The result indicated that IFN-τ, especially at 12 h, could regulate the composition of gut microbiota associated with *Pediococcus*, Staphylococcus, and *Enterorhabdus* in E. coli-induced mouse endometritis.

We speculated that changes in metabolic pathways might be at least partially affected by the gut microbiome of mice with endometritis. Subsequently, metabolomics analysis confirmed this conjecture that significant metabolites including *O*-phospho-l-serine, ureidoisobutyric acid, ADP-ribose, GDP-l-fucose, glutathione oxidized, and carnitine C_13:0_ were downregulated effectively by IFN-τ treatment at 12 h. It is reported that the expression level of these metabolites is strongly associated with the development of many inflammatory diseases, which is consistent with the results of this study (28–30). These metabolites are involved in metabolic pathways such as cysteine and methionine metabolism, pyrimidine metabolism, and arachidonic acid metabolism (31). Metabolic stress leads to inflammation, and inflammation itself will destroy the metabolic balance of the body. Therefore, metabolic disorder and inflammatory reaction may form a vicious circle (32). Therefore, these results indicated that IFN-τ participated in these metabolic pathways to interfere with the inflammatory response. Moreover, the analysis of correlations between gut microbiota and metabonomics of uterine tissue showed that changes in metabolic pathways might be affected by gut microbiota, such as *Enterorhabdus* in mouse endometritis. Research showed that lower levels of *Enterorhabdus* in the gut indicated improved gut health in mice (33) conclusively, IFN-τ alleviated the damage in mouse endometritis induced by E. coli and improved the endometrial tissue barrier. Its functional mechanism may be to reduce the abundance of *Enterorhabdus* in the gut microbiota, affecting the expression level of important metabolites in uterine tissue and thus playing an anti-inflammatory role.

## MATERIALS AND METHODS

Recombinant bovine tau interferon (IFN-τ; high-performance liquid chromatography [HPLC], >97%) was purchased from Creative Bioarray (NY, USA). The antibody and electron microscope fixative were purchased from Servicebio (Wuhan, China).

### Animal treatment and experimental groups.

Mice were purchased from the Laboratory Animal Center of Zhejiang University (Hangzhou, China). A total of 50 BALB/c female mice (8 weeks old) were used in the study. The mice were fed freely. Before the experiment, the mice were fed under dark-light circulation for 12 h at 24°C ± 1°C and 60% humidity and housed in separate cages. All experimental protocols and procedures were approved by the Institutional Animal Care and Use Committee of Zhejiang University.

The mice were classified at random into the following 5 groups of 10 mice each to induce the endometritis model: control group (CG), E. coli group (ECO), and IFN-τ (8 μg/kg of body weight, 6, 12, and 24 h) groups (IF6, IF12, and IF24, respectively). The method of modeling endometritis was described earlier (34). Briefly, the mice were anesthetized with sodium pentobarbital, and each uterus was infused with E. coli (1 × 10^6^ CFU/mL) to cause endometritis. After 24 h of perfusion, IFN-τ groups received an intraperitoneal injection of IFN-τ at 6, 12, and 24 h, respectively. The CG received the same amount of saline intraperitoneally. Finally, mice were euthanized with CO_2_ inhalation, and the uterus tissues and feces samples were harvested and stored at −80°C.

### High-throughput 16S rRNA gene sequencing.

The Tiangen fecal DNA kit (Beijing, China) was used to extract microbial genomic DNA from mouse fecal samples. Universal primers (forward [F], Illumina_uni_sequence-Read1_sequnce_GTGCCAGCMGCCGCGGTAA; reverse [R], Illumina_uni_sequence_read2_sequence_GGACTACHVGGGTWTCTAAT) were used for PCR amplification of bacterial 16S rRNA gene V3-V4 regions. The PCR system was Platinum HiFi PCR mix (50 μL), including 25 μL of Phusion high-fidelity (HF) PCR master mix with HF buffer, 3 μL of F/R primers (10 μM), 10 μL template DNA, and 6 μL double-distilled water (ddH_2_O). The PCR products were detected by 1% agarose gel electrophoresis. After the library was successfully built, it was quantified via the PicoGreen double-stranded DNA (dsDNA) assay kit (Invitrogen, Carlsbad, CA, USA) and purified using AMPure XP beads (Beckman Coulter, Indianapolis, IN). Sequencing was performed on the Illumina NovaSeq 6000 paired-end 2- by 150-bp sequencer. These sample readings obtained by Vsearch v2.4.4 software were first spliced according to the overlap relationship, while controlling and filtering the sequence quality. Sequence analysis was performed by Vsearch v2.4.4 software. Classification information was annotated with the SILVA database based on the Mothur algorithm (35).

Subsequently, α diversity (Chao1 and Simpson diversity indices, etc.), β diversity (principal-coordinate analysis [PCoA]), and other analyses were based on output normalized data and calculated using QIIME and R software (v3.2.0). The Student *t* test and Monte Carlo permutation test with 1,000 permutations were used to determine the difference of UniFrac distance between pairs of groups, and box-and-whisker diagrams were used to visualize it. The R package “vegan” was used to evaluate the significance of structural differentiation of microbiota among groups through PERMANOVA (permutational multivariate analysis of variance). Venn diagrams were generated using the R package “VennDiagram” to visualize shared and unique operational taxonomic units (OTUs) between samples or groups based on the occurrence of OTUs across samples/groups, regardless of their relative abundance. Using the default parameters, LEfSe (linear discriminant analysis effect size) was used to detect taxa with rich differences between groups. Random forest analysis was used for distinguishing different groups of samples using the R package “randomForest” with all default settings and 1,000 trees. The generalization error was estimated using 10-fold cross-validation. The expected “baseline” error was also included, which was obtained by a classifier that simply predicts the labels of the most common classes. Cooccurrence analysis was conducted through calculating Spearman’s rank correlation among dominant taxa. Cytoscape was used to visualize the correlation of |RHO| of >0.6 and *P* of <0.01 as a cooccurrence network. Relying on high-quality sequences, PICRUSt (*P*hylogenetic *I*nvestigation of *C*ommunities by *R*econstruction of *U*nobserved *St*ates) predicted microbial function. The output files were further analyzed using the Statistical Analysis of Metagenomic Profiles (STAMP) package v2.1.3. FAPROTAX is a database that draws prokaryotic branches to established metabolic or other ecologically related functions (36, 37).

### Uterine tissue metabolomics. (i) Tissue sample preparation.

A cold steel ball was added to 50 mg of sample, and the mixture was homogenized at 30 Hz for 3 min. The internal standard extract and 1 mL of 70% methanol were added to the homogeneous centrifuge tube, spun for 5 min, and centrifuged at 12,000 rpm for 10 min at 4°C. After centrifugation, the 400-μL supernatant was pumped into the corresponding Eppendorf tube, preserved overnight in a −20°C freezer, and then centrifuged at 12,000 revolutions/min for 3 min at 4°C, and 2 mL of supernatant was taken into the inner tank of the corresponding injection vial for onboard analysis.

### (ii) Metabolomics data analysis.

An LC-ESI-MS/MS system (ultrahigh-performance liquid chromatography [UPLC], ExionLC AD, https://sciex.com.cn/; MS, QTrap system, https://sciex.com/) was used to analyze sample extracts. The analytical conditions were as follows for UPLC: column, Waters Acquity UPLC HSS T3 C_18_ (1.8 μm, 2.1 mm by 100 mm); column temperature, 40°C; flow rate, 0.4 mL/min; injection volume, 2 μL; solvent system, water (0.1% formic acid)-acetonitrile (0.1% formic acid); gradient program, 95:5 (vol/vol) at 0 min, 10:90 (vol/vol) at 10.0 min, 10:90 (vol/vol) at 11.0 min, 95:5 (vol/vol) at 11.1 min, 95:5 (vol/vol) at 14.0 min. Unsupervised principal-component analysis (PCA) was conducted by prcomp, a statistical function in R. Before unsupervised PCA, the unit variance of the data was scaled. VIP of ≥1 and absolute log2FC (fold change) of ≥1 determined the significantly regulated metabolites between groups. The KEGG compound database was used to label identified metabolites (http://www.kegg.jp/kegg/compound/), and then the annotated metabolites were mapped to the KEGG pathway database (http://www.kegg.jp/kegg/pathway.html). Then, pathways with significant regulation of metabolites were mapped to the metabolite enrichment analysis, and their significance was determined by the *P* value of the hypergeometric test.

### Combined analysis of microbiome and metabolomics.

Disordered gut microbial genera were obtained from 16S rRNA sequencing, and Pearson correlation analysis was carried out between differential metabolites obtained from tissue metabolomics. The correlation coefficients were |r| of >0.70 and *P* of <0.05. Finally, significantly correlated metabolites and gut microbe genera were obtained and shown as a hot spot map (*, *P* < 0.05; **, *P* < 0.01). Red shows a positive correlation while green shows a negative correlation.

### Histopathological analysis.

Mouse uterine tissue was obtained and fixed in 10% formalin for later histopathological analysis. In brief, dehydration with different concentrations of ethanol, paraffin embedding, sectioning, and hematoxylin and eosin (H&E) staining were used to visualize morphological changes of uterine tissue under light microscopy (Olympus, Japan).

### MPO assay.

The uterus tissues of each group were collected, and phosphate-buffered saline (PBS) was used to homogenate 100 mg of tissues. These supernatants were analyzed by a commercial kit (myeloperoxidase [MPO]; Jiangcheng Biotechnology, Nanjing, China) according to the instructions of the supplier and using the spectrophotometry method for detection at 460 nm.

### TEM analysis.

The tissue block was harvested, and PBS was used to wash the blood, hair, etc. The washed tissue blocks were fixed with electron microscope fixative (Servicebio, Wuhan, China) immediately at room temperature for 2 h and then stored at 4°C. After dehydration of the graded ethanol series and then isoamyl acetate for 15 min, a carbon sticker was used to attach the specimens to a metal stub and sputter coated with gold for 30 s. Finally, images were observed and taken with a transmission electron microscope (TEM).

### Immunofluorescence staining analysis.

The immunofluorescence staining assay was performed following the manufacturer’s directions. Briefly, uterine tissues about 1 cm in size were obtained, fixed in 10% formaldehyde, and then embedded with paraffin. These slices were permeated with PBS, and 0.3% Triton X-100 (Sigma, MO, USA) and 10% bovine serum albumin (BSA) were added. Tissue sections were incubated with specific antibodies and Cy3 secondary antibodies for 12 h at 4°C. Finally, the protein was determined and immobilized using mounting medium supplemented with 4′,6-diamidino-2-phenylindole (DAPI) (Servicebio, Wuhan, China). All of the slices were observed by fluorescence microscopy.

### Statistical analysis.

Data analysis used SPSS software. Statistics were represented as the mean ± standard error of the mean (SEM) from three individual experiments. Data were analyzed by one-way analysis of variance (ANOVA) and the Student *t* test. Significance was deemed at a *P* value of <0.05.

### Data availability.

The data of this study are available from the corresponding author upon request.
