# Supplementary material for: Uterine Tissue Metabonomics Combined with 16S rRNA Gene Sequencing To Analyze the Changes of Gut Microbiota in Mice with Endometritis and the Intervention Effect of Tau Interferon
Source: Microbiol Spectr. 2023 Apr 17;11(3):e00409-23. doi: 10.1128/spectrum.00409-23 (PMC10269590; doi:10.1128/spectrum.00409-23)
Supplement: Supplemental file 1 — Supplemental material. Download spectrum.00409-23-s0001.pdf, PDF file, 0.7 MB [file spectrum.00409-23-s0001.pdf]

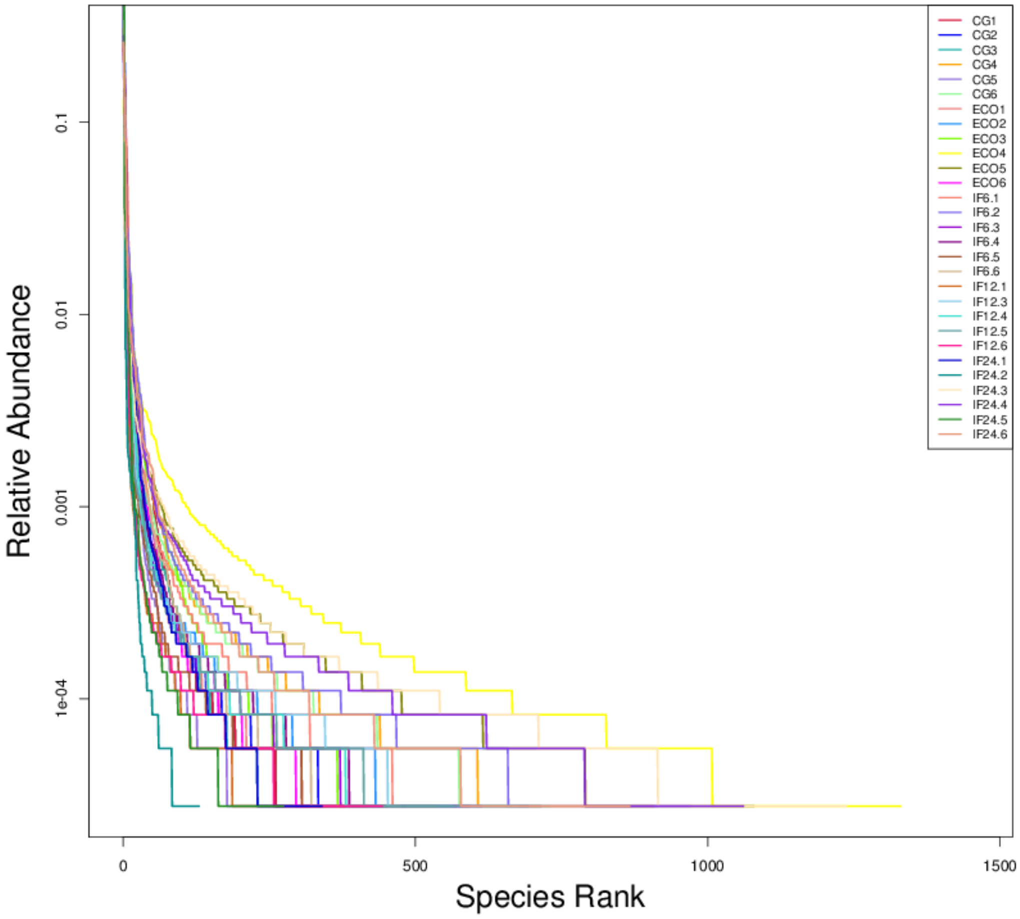

Supplementary Figure S1 Species accumulation curve.

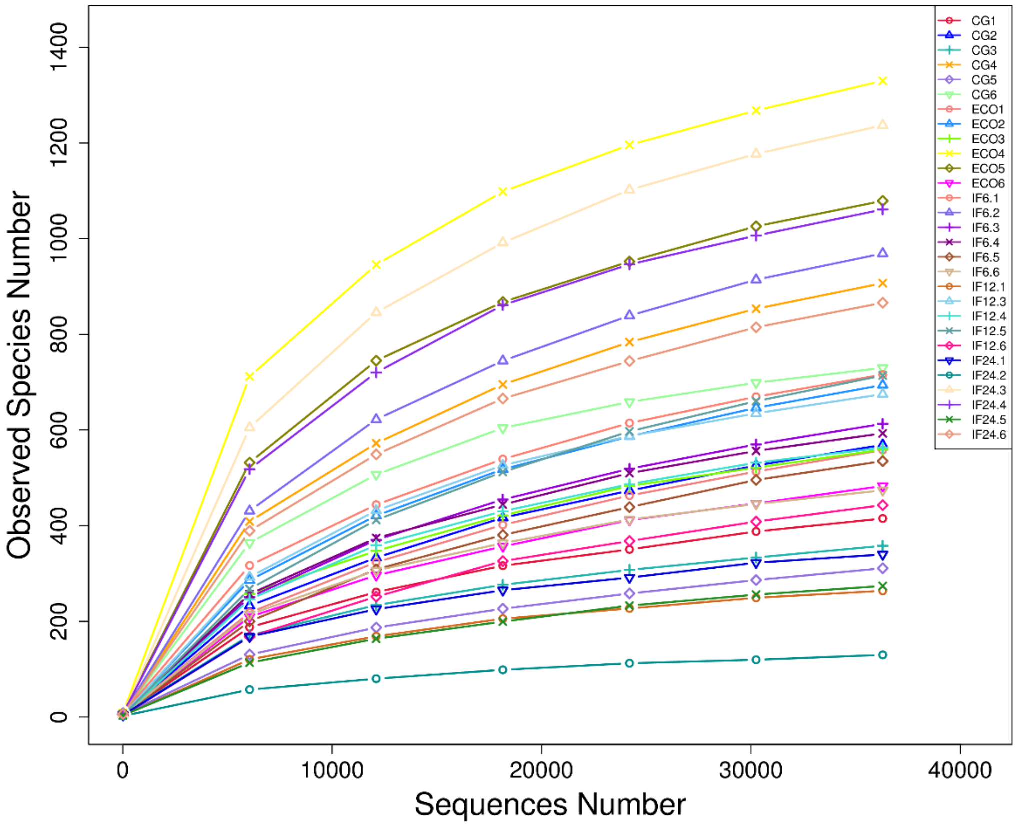

Supplementary Figure S2 Rarefaction Curve.

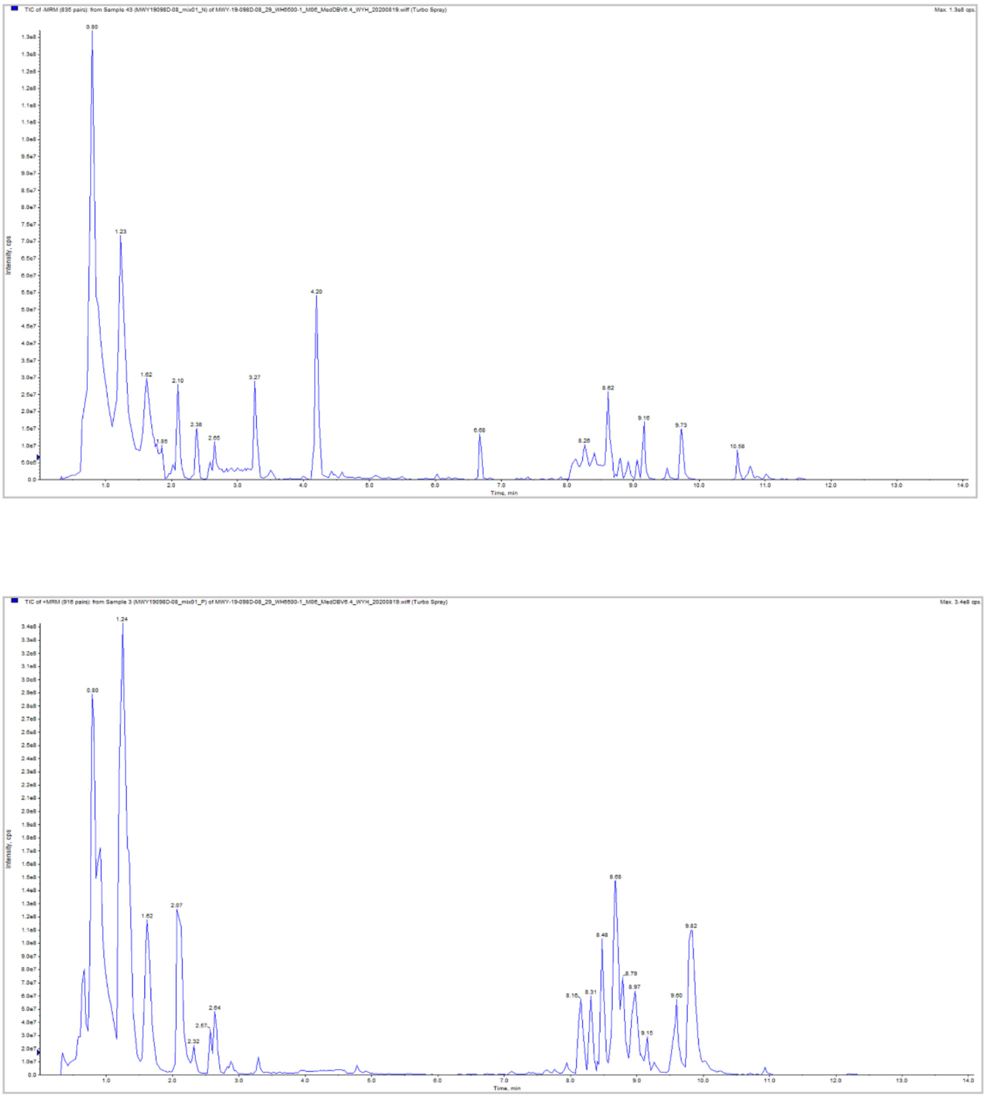

Supplementary Figure S3 The total ion chromatogram (TIC) profiles of the uterine tissue.

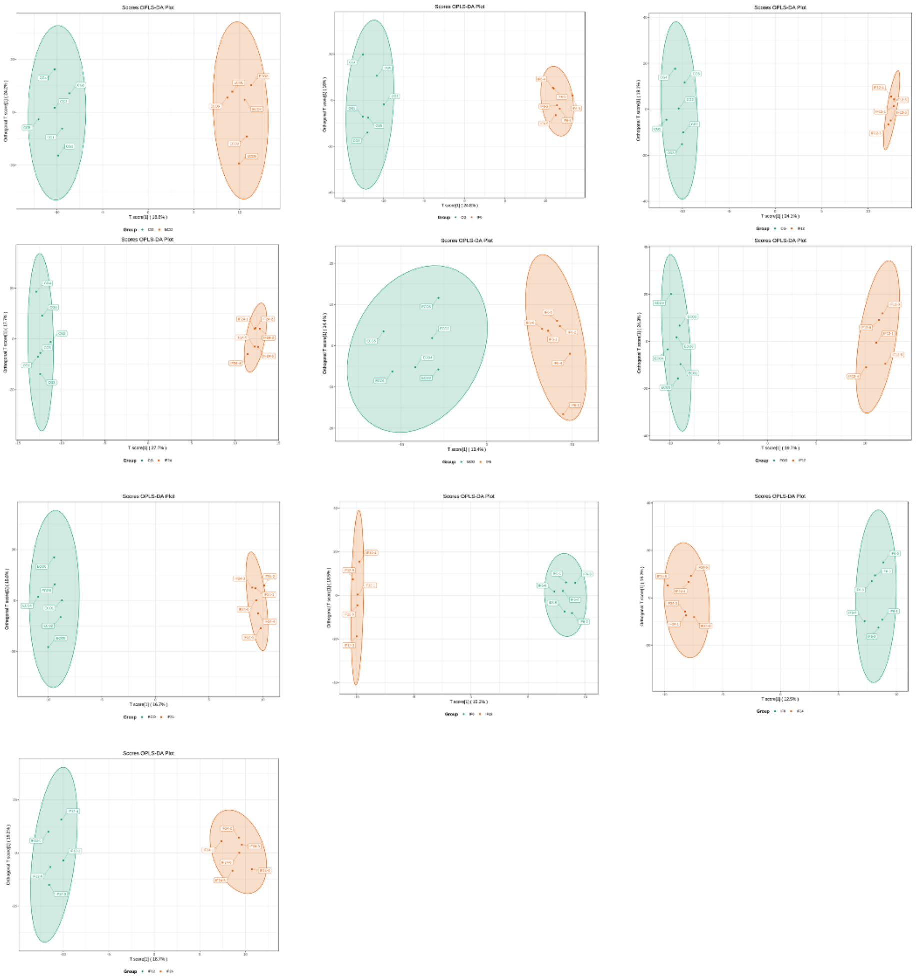

Supplementary Figure S4 OPLS-DA score plots of Metabolites.
